# Synergistic anti‐depressive effect of combination treatment of Brexpiprazole and selective serotonin reuptake inhibitors on forced swimming test in mice

**DOI:** 10.1002/npr2.12316

**Published:** 2023-01-17

**Authors:** Naoki Amada, Tsuyoshi Hirose, Mikio Suzuki, Yusuke Kakumoto, Takashi Futamura, Kenji Maeda, Tetsuro Kikuchi

**Affiliations:** ^1^ Department of CNS Research Otsuka Pharmaceutical Co., Ltd. Tokushima Japan; ^2^ Department of Lead Discovery Research Otsuka Pharmaceutical Co., Ltd. Tokushima Japan; ^3^ Pharmaceutical Division Otsuka Pharmaceutical Co., Ltd. Tokushima Japan

**Keywords:** Brexpiprazole, forced swimming test, major depressive disorder, psychiatric disorder, selective serotonin reuptake inhibitor

## Abstract

**Aim:**

Selective serotonin reuptake inhibitors (SSRIs) are used to treat major depressive disorder (MDD) and other psychiatric disorders (e.g., obsessive compulsive disorder, social anxiety disorder, and panic disorder). In MDD treatment, SSRIs do not show remission in approximately 30% of patients, indicating a need for a better treatment option. Forced swimming test (FST) is a behavioral assay to evaluate depression‐like behavior and antidepressant efficacy in rodents. In the present study, we evaluated the combination effect of brexpiprazole with SSRIs on FST in mice, in order to investigate their synergistic effect.

**Methods:**

Brexpiprazole (0.003 mg/kg) was intraperitoneally injected to mice 15 min before testing. Escitalopram (10 mg/kg), fluoxetine (75 mg/kg), paroxetine (10 mg/kg), or sertraline (15 mg/kg) were orally administered to mice 60 min before testing. Then, the mice were placed in water and immobility time was measured. Data from animals treated with escitalopram, fluoxetine, paroxetine, and sertraline were pooled as SSRI‐treated group data.

**Results:**

Combination treatment of brexpiprazole with SSRIs reduced immobility time in FST more than vehicle or each single treatment. A significant interaction effect was confirmed in the combination of brexpiprazole and SSRIs (*p* = 0.0411).

**Conclusion:**

Efficacy of adjunctive brexpiprazole has already been demonstrated in clinical trials in MDD patients not adequately responding to antidepressants including escitalopram, fluoxetine, paroxetine, and sertraline. The synergistic antidepressant‐like effect of brexpiprazole with SSRIs found in this study supports the already known clinical findings.

## Abstract 2

Synergistic antidepressant‐like effect of brexpiprazole with selective serotonin reuptake inhibitors was confirmed on the forced swimming test in mice. The combination treatment reduced immobility time in the forced swimming test more than vehicle or each single treatment.

## Graphical abstract

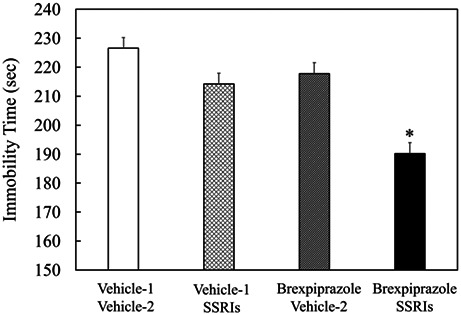

## 1 INTRODUCTION

Major depressive disorder (MDD) is a world public health problem, which causes loss of productivity and increased mortality including suicide.^1^ Selective serotonin reuptake inhibitors (SSRIs) are used to treat MDD and several other psychiatric disorders (e.g., obsessive compulsive disorder, social anxiety disorder, and panic disorder).^2^ In the case of MDD treatment, it is considered that approximately 30% of patients do not respond to antidepressant treatment including SSRIs.^3^ For patients with inadequate response to antidepressant treatment, evidence to show effectiveness of atypical antipsychotics as adjunctive treatment has been recognized.^4^, ^5^ However, atypical antipsychotics are associated with side effects such as weight gain, sedation, and akathisia. There is still a need for an effective, safe, and well‐tolerated atypical antipsychotic for treatment of MDD patients.^5^

Brexpiprazole is a serotonin–dopamine activity modulator developed as a novel treatment for psychiatric disorders.^6^, ^7^ Brexpiprazole is approved for the treatment of adults with schizophrenia in countries including the United States (US), Canada, Australia, Japan, and the European Union.^5^ Brexpiprazole is also approved as an adjunctive therapy to antidepressants for adults with MDD in the United States and several other countries.^5^ Brexpiprazole acts as a partial agonist at serotonin 5‐HT_1A_ and dopamine D_2_ receptors, and as an antagonist at 5‐HT_2A_ and noradrenaline α_1B/2C_ receptors.^6^ Its risk for catalepsy (extrapyramidal symptoms) and hyperprolactinemia in animals is lower than the risk observed for risperidone.^6^, ^7^ Furthermore, we have shown in an animal study that brexpiprazole has a low risk of D_2_ receptor supersensitivity after a repeated administration, indicative of a low propensity to cause dopamine supersensitivity psychosis and tardive dyskinesia in the course of long‐term treatment.^8^ These tell us that brexpiprazole is likely a well‐tolerated antipsychotic drug.

Forced swimming test (FST) is used to evaluate depression‐like behavior and antidepressant efficacy in rodents, in which immobility is thought to reflect behavioral despair in rodents.^9^, ^10^ In order to demonstrate synergistic antidepressant‐like effect in animals, we evaluated combination effect of brexpiprazole with SSRIs, using each ineffective dose based on unpublished in‐house data, on immobility time in FST in mice. For this combinatory study, we selected SSRIs (escitalopram, fluoxetine, paroxetine, and sertraline) already approved for treatment of MDD.

## 2 MATERIALS AND METHODS

### 2.1 Animals

Male ddY mice (Japan SLC, Inc., 5 to 6 weeks old) were used. They were group‐housed in individual cages with water and food (Oriental Yeast Co, Ltd., Tokyo, Japan) supplied ad libitum, and maintained under artificial lighting between 7:00 am to 7:00 pm. The room temperature and humidity were maintained at 23 ± 2°C and 60 ± 10%, respectively.

### 2.2 Treatment

Brexpiprazole and SSRIs (escitalopram, fluoxetine, paroxetine, and sertraline) were synthesized at Otsuka Pharmaceutical Co., Ltd. For evaluation of the combination treatment, in order to avoid increase of per os (p.o.) administration volume and any influence on intestinal absorption, different administration routes were used for brexpiprazole (intraperitoneal: i.p.) and SSRIs (p.o.), which also enabled two ways (i.p. and p.o.) of vehicle treatment for the combination study. Brexpiprazole was dissolved in 1% lactic acid saline (vehicle‐1) at the concentration of 0.0003 mg/ml. Vehicle‐1 and brexpiprazole were injected to mice at a volume of 10 ml/kg (i.p.), 15 min before testing. Escitalopram, fluoxetine, paroxetine, and sertraline were suspended in 5% (w/v) gum arabic‐distilled water solution (vehicle‐2) at the concentration of 6 mg/ml, 7.5 mg/ml, 1 mg/ml, and 1.5 mg/ml, respectively. Vehicle‐2 and these SSRIs were administered to mice at a volume of 10 ml/kg (p.o.), 60 min before testing. Ineffective doses of brexpiprazole and the SSRIs, based on unpublished in‐house data, were used for the combination study. Our in‐house data show that the plasma concentration of brexpiprazole is higher in i.p. than p.o. administration (data not shown). As a result, the i.p. dose of brexpiprazole used in this study was low.

### 2.3 Forced swimming test

Animals were assigned to four groups (Table 1). FST was conducted according to previous reports with slight modification.^9^, ^11^, ^12^ After the administration of brexpiprazole and either of SSRIs, each mouse was placed into an acrylic cylinder (25 cm high × 9 cm inner diameter) filled with water (23 ± 1°C) up to 10 cm from the bottom of cylinder for 6 min. Then, the mobility time of the mouse was measured using SCANET (Melquest Co., Ltd., Toyama, Japan), and the immobility time was determined during last 4 min.

**TABLE 1:** Treatment

Group	Treatment‐1 (i.p. injection)	Treatment‐2 (p.o. administration)	*N*
1	Vehicle‐1	Vehicle‐2	37
2	Vehicle‐1	SSRIs	37
3	Brexpiprazole	Vehicle‐2	37
4	Brexpiprazole	SSRIs	37

*Note*: Vehicle‐1: 1% lactic acid/saline. Vehicle‐2: 5% (w/v) gum arabic‐distilled water solution. Vehicle‐1 and brexpiprazole (0.003 mg/kg) were i.p. injected to mice. SSRIs included escitalopram (60 mg/kg), fluoxetine (75 mg/kg), paroxetine (10 mg/kg), and sertraline (15 mg/kg). Vehicle‐2 and these SSRIs were p.o. administered to mice.

### 2.4 Statistical analysis

Data from escitalopram, fluoxetine, paroxetine, and sertraline treatments were pooled as results from SSRI‐treated animals. The synergistic effects of brexpiprazole and SSRIs were assessed by a factorial ANOVA with the test‐day as a covariate. The presence or absence of synergistic effects of the combination was evaluated using interaction effects. The statistical analysis was conducted using SAS Software for Windows, Release 9.4 (SAS Institute Japan Ltd., Tokyo, Japan). The difference was considered statistically significant, when *p* value was <0.05.

## 3 RESULTS

**FIGURE 1:**
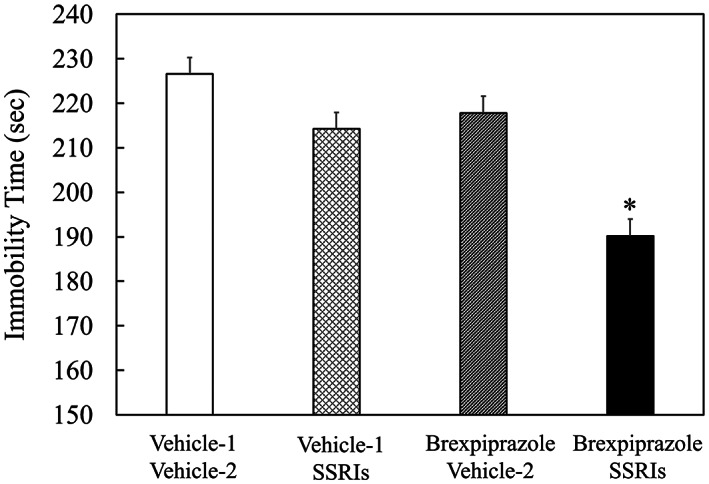
Forced swimming test results. Mice were placed in water for 6 min and immobility time was determined during last 4 min. Data are expressed as mean ± SEM. Vehicle‐1 (1% lactic acid/saline) and brexpiprazole (0.003 mg/kg) were i.p. injected to mice. Vehicle‐2 (5% (w/v) gum arabic‐distilled water solution) and SSRIs were p.o. administered to mice. SSRIs included escitalopram (60 mg/kg), fluoxetine (75 mg/kg), paroxetine (10 mg/kg), and sertraline (15 mg/kg). The interaction between brexpiprazole and SSRIs (*p* = 0.0411) was assessed by a factorial ANOVA with the test‐day as a covariate (*p = 0.0411).

Group 4 (brexpiprazole + SSRIs) animals showed less immobility time compared to group 1 (vehicle‐1 + vehicle‐2), group 2 (vehicle‐1 + SSRIs), and group 3 (brexpiprazole + vehicle‐2) animals (Figure 1 and Table 2). There was a significant interaction effect in the combination of brexpiprazole and SSRIs (*p* = 0.0411). This indicates that brexpiprazole shows synergistic effect with SSRIs.

**TABLE 2:** Forced swimming test results

Group		Immobility time (s)
1	Vehicle‐1 + Vehicle‐2	226.6 ± 3.7
2	Vehicle‐1 + SSRIs	214.3 ± 3.7
3	Brexpiprazole + Vehicle‐2	217.8 ± 3.7
4	Brexpiprazole + SSRIs	190.2 ± 3.7^a^

*Note*: Data are expressed as mean ± SEM.

^a^ A significant interaction between brexpiprazole and SSRIs was confirmed by a factorial ANOVA with the test‐day as a covariate (*p* = 0.0411).

## 4 DISCUSSION

Brexpiprazole is approved as an adjunctive therapy to antidepressants for the treatment of adults with MDD in the United States and other countries.^5^ In its clinical trials, the efficacy of adjunctive brexpiprazole was demonstrated in MDD patients who did not respond adequately to antidepressant treatments including escitalopram, fluoxetine, paroxetine, and sertraline.^13^, ^14^, ^15^

For determination of synergistic antidepressant‐like effects of brexpiprazole with SSRIs in animals, in this study we selected and used SSRIs (escitalopram, fluoxetine, paroxetine, and sertraline) already approved for treatment of MDD. We demonstrated a significant synergistic effect of brexpiprazole and the SSRIs in mice, confirming clinical trial findings for the adjunctive effect of brexpiprazole to antidepressants.

Brexpiprazole acts as a partial agonist at 5‐HT_1A_ and D_2_ receptors, and an antagonist at 5‐HT_2A_ and α_1B/2C_ receptors.^6^ It has been hypothesized that the receptor binding and functional profile of brexpiprazole at these receptors may make brexpiprazole a better choice for adjunctive treatment of MDD than other products commonly used when there is insufficient response to antidepressants alone^15^ because of the known antidepressant and anxiolytic effects of 5‐HT_1A_ receptor partial agonists and 5‐HT_2A_ receptor antagonists.^16^ Antagonistic action on α_1_ adrenergic receptors is considered to have potential therapeutic effect in MDD.^17^ Overexpression of theα_2C_ adrenergic receptor increased immobility time in FST in mice, while knockout or antagonists of the receptor decreased.^18^, ^19^ Therefore, brexpiprazole has a favorable in vitro functional profile to show antidepressant effects. Although the contribution of D_2_ receptor partial agonist activity on MDD treatment is not well known, brexpiprazole's D_2_ receptor partial agonist activity may also be a potential contributor of its antidepressant effects.

Moreover, brexpiprazole was well tolerated in its clinical trials in MDD patients.^13^, ^14^, ^15^ It has been known that administration of not only typical antipsychotics but also atypical antipsychotics have a risk to cause tardive dyskinesia.^20^, ^21^ Long‐term D_2_ receptor blockade can evoke dopamine D_2_ receptor supersensitivity both in animals and humans, which is considered to be a possible cause of tardive dyskinesia.^22^, ^23^, ^24^ We previously reported that brexpiprazole had a low risk to induce D_2_ receptor supersensitivity in rats after repeated administration,^8^ indicative of a low propensity to cause tardive dyskinesia. Indeed, no clinically relevant findings related to extrapyramidal symptoms were reported in a 52‐week clinical study evaluating the safety and tolerability of brexpiprazole as adjunctive therapy in adult MDD patients.^5^ In addition, according to Citrome's report, other antipsychotics approved as adjunctive agents for the treatment of MDD (aripiprazole and quetiapine extended release [ER]) and olanzapine‐fluoxetine combination approved as a combination agent for the treatment of treatment‐resistant MDD had a higher incidence of akathisia (aripiprazole) or sedation/somnolence (quetiapine ER and olanzapine‐fluoxetine combination) than placebo treatment.^25^ Data in the report indicate less incidence of akathisia with brexpiprazole than with aripiprazole, and for sedation/somnolence than quetiapine ER and olanzapine‐fluoxetine combination.^25^

Taken together with our present data and the known clinical data, brexpiprazole has a potential to be a better therapeutic choice for adjunctive treatment of MDD patients.

## AUTHOR CONTRIBUTIONS

NA wrote the paper. TH conceived and designed the study, performed experiments. YK conducted and was responsible for statistics. KM, TF, and TK conceived the study, and reviewed the paper. MS reviewed the paper. All authors contributed to finalizing the paper and had final responsibility for the decision to submit for publication, took part in either drafting and/or revising the paper, and approved the final version of the paper.

## FUNDING INFORMATION

Otsuka Pharmaceutical Co., Ltd.

## CONFLICT OF INTEREST

TH retired from Otsuka Pharmaceutical Co., Ltd. All the other authors are employees and own stock of Otsuka Holdings Co., Ltd.

## ETHICAL APPROVAL

Approval of the Research Protocol by an Institutional Reviewer Board

The experimental procedure in this study was approved and conducted in accordance with Guidelines for Animal Care and Use in Otsuka Pharmaceutical Co, Ltd.

Informed Consent: N/A.

Registry and the Registration no. of the Study/trial: N/A.

Animal Studies: The care and handling of the animals was in accordance with “Guidelines for Animal Care and Use in Otsuka Pharmaceutical Co, Ltd.”

## Supporting information


Data S1

## Data Availability

All data are available in the Data S1.
