# Correction to “Synergistic anti‐depressive effect of combination treatment of Brexpiprazole and selective serotonin reuptake inhibitors on forced swimming test in mice”

**DOI:** 10.1002/npr2.12474

**Published:** 2024-08-28

## Abstract

AmadaN , HiroseT , SuzukiM , KakumotoY , FutamuraT , MaedaK , et al. Synergistic anti‐depressive effect of combination treatment of Brexpiprazole and selective serotonin reuptake inhibitors on forced swimming test in mice. Neuropsychopharmacol Rep. 2023;43:132–136.36649966 10.1002/npr2.12316PMC10009414

In the ‘Methods’ section of the Abstract, the second sentence is as follows:

“Escitalopram (10 mg/kg), fluoxetine (75 mg/kg), paroxetine (10 mg/kg), or sertraline (15 mg/kg) were orally administered to mice 60 min before testing.”

The amount listed for escitalopram is incorrect. It should be: 60 mg/kg.

We apologize for this error.

Amada N , Hirose T , Suzuki M , Kakumoto Y , Futamura T , Maeda K , et al. Synergistic anti‐depressive effect of combination treatment of Brexpiprazole and selective serotonin reuptake inhibitors on forced swimming test in mice. Neuropsychopharmacol Rep. 2023;43:132–136.36649966 10.1002/npr2.12316PMC10009414

In the ‘Methods’ section of the Abstract, the second sentence is as follows:

“Escitalopram (10 mg/kg), fluoxetine (75 mg/kg), paroxetine (10 mg/kg), or sertraline (15 mg/kg) were orally administered to mice 60 min before testing.”

The amount listed for escitalopram is incorrect. It should be: 60 mg/kg.

We apologize for this error.

